# Cognitive decline, and quality of life: evidence from community-dwelling older adults in the United Arab Emirates

**DOI:** 10.3389/fragi.2026.1822042

**Published:** 2026-05-29

**Authors:** Wegdan Bani Issa, Dana N. Abdelrahim, Hadia Radwan, Manal Awad, Farah Naja, Roba Saqan, Tamer Shousha, Heba Khalil, Ayat Nashwan, Adam Ibrahim, Hajar Ibrahim, Latifa Alsaad, Heba Hijazi, Alham Al-Sharman, Rachel Rossiter, Jassim Alhammadi, Mohamad Alameddine

**Affiliations:** 1 Nursing Department, College of Health Sciences, University of Sharjah, Sharjah, United Arab Emirates; 2 Research Institute for Medical and Health Sciences, University of Sharjah, Sharjah, United Arab Emirates; 3 Department of Clinical Nutrition and Dietetics, College of Health Sciences, University of Sharjah, Sharjah, United Arab Emirates; 4 Department of Orthodontics, Pediatric and Community Dentistry, College of Dental Medicine, University of Sharjah, Sharjah, United Arab Emirates; 5 Center of Excellence for Healthy Aging, University of Sharjah, Sharjah, United Arab Emirates; 6 Department of Physiotherapy, College of Health Sciences, University of Sharjah, Sharjah, United Arab Emirates; 7 Department of Physical Therapy for Musculoskeletal Disorders and its Surgery, Faculty of Physical Therapy, Cairo University, Cairo, Egypt; 8 Department of Sociology, College of Arts, Humanities, and Social Sciences, University of Sharjah, Sharjah, United Arab Emirates; 9 Department of Sociology and Social Work, Faculty of Art, Yarmouk University, Irbid, Jordan; 10 College of Medicine, University of Sharjah, Sharjah, United Arab Emirates; 11 Al Kuwaiti Hospital, Internship Program, Emirate Health Services, Abu Dhabi, United Arab Emirates; 12 Research and Graduate Studies Department, Mohammed Bin Rashid University of Medicine and Health Sciences, Dubai Health, Dubai, United Arab Emirates; 13 Department of Health Care Management, College of Health Sciences, University of Sharjah, Sharjah, United Arab Emirates; 14 Department of Health Management and Policy, Faculty of Medicine, Jordan University of Science and Technology, Irbid, Jordan; 15 School of Rural Medicine, Faculty of Science and Health, Charles Sturt University, Orange, NSW, Australia; 16 Sharjah Social Services Department, Sharjah, United Arab Emirates

**Keywords:** cognitive function, montreal cognitive assessment, older adults, quality of life, UAE

## Abstract

**Background:**

Cognitive function and quality of life (QoL) are closely linked in older adults, with accumulating evidence suggesting that cognitive performance is an important determinant of overall perceived QoL in older adults.

**Objectives:**

To examine cognitive function and its association with multidimensional QOL among adults aged ≥60 years in the United Arab Emirates (UAE) and identify sociodemographic and lifestyle correlates.

**Methods:**

A cross-sectional study of 606 community-dwelling older adults from the UAE (55.4% male; mean age 67.2 ± 5.8 years) assessed cognitive function (MoCA) and QOL (OPQoL-Brief). Associations were analyzed using correlation and multiple linear regression (p < 0.05) between the sociodemographic factors and cognitive function (MOCA) to QOL.

**Results:**

Participants aged 60–87 years were predominantly Emirati (84.2%), married (77.6%), and living with family (96.7%). Cognitive impairment was present in 69.3% (40.9% mild, 24.8% moderate, 3.6% severe), with the lowest abstraction scores. Mean QOL was 4.21 ± 0.51/5, lowest in social engagement. Cognitive impairment independently predicted lower QOL (β = 0.138; 95%CI: 0.043–0.232; p = 0.001), along with female sex, older age, lower education or income, unmarried status, living alone, and low physical activity.

**Conclusion:**

Cognitive function is a key determinant of QOL, supporting integrated, person-centered aging strategies in the UAE.

## Introduction

Population aging is one of the most consequential demographic transitions of the twenty-first century. Improvements in healthcare, living conditions, and life expectancy have led to a rapid expansion of the global population aged 60 years and older. The World Health Organization (WHO) estimates that the number of older adults worldwide will increase from approximately 1.1 billion in 2023 to nearly 1.4 billion by 2030, reflecting a substantial shift in population structure toward later life (World Health Organization, 2025a). This demographic transition places increasing demands on health systems, as older adults experience a higher burden of chronic and age-related conditions requiring sustained medical, social, and long-term care (Hajizadeh et al., 2025).

Among these conditions, cognitive decline represents a particularly important public health concern because of its implications for functional ability and independence. Dementia affects more than 55 million people globally, and prevalence is expected to rise substantially over the coming decades, largely driven by population aging (World Health Organization, 2025b). Beyond diagnosed dementia, age-related cognitive decline has been recognized as a key contributor to disability and loss of autonomy in later life. The 2024 update of the Lancet Commission on dementia emphasizes that cognitive impairment should be viewed not only as a neurological condition, but also as a determinant of everyday functioning and wellbeing across the aging trajectory (Livingston et al., 2024).

Healthy aging is increasingly defined by the ability to maintain functional capacity and quality of life (QOL) rather than longevity alone. QOL is a multidimensional and subjective construct encompassing physical health, psychological wellbeing, level of independence, social relationships, and interaction with the surrounding environment (World Health Organization, 2025c). In older adults, cognitive functioning plays a central role in shaping these domains by influencing decision-making, autonomy, social participation, and engagement in daily activities. Accordingly, QOL offers a meaningful and measurable framework for understanding the real-world impact of cognitive decline in later life.

A growing body of recent evidence demonstrates a consistent association between cognitive function and QOL among older adults. Systematic reviews and meta-analyses indicate that even mild cognitive impairment is associated with significantly poorer QOL, independent of a formal dementia diagnosis (Gopalakrishnan et al., 2024). Longitudinal population-based studies further show that declines in cognitive function are associated with reduced independence in activities of daily living, increased psychological distress, and lower overall life (Karam et al., 2024). Together, these findings highlight cognitive health as a central determinant of QOL and reinforce its relevance within healthy aging strategies.

In sync with global demographic trends, the United Arab Emirates is experiencing early population aging. Although older adults currently represent a relatively small proportion of the total population, this proportion is increasing, particularly among those aged 65 years and above, with projections indicating continued growth and important implications for healthcare planning and service delivery (Sheikh and Munawar, 2024; United Nations, 2024). Interpretation of aging trends in the UAE requires caution, as population structure is strongly influenced by a large proportion of young expatriate workers, which may obscure aging patterns when aggregate population data are considered. Nevertheless, cognitive decline among older adults in the UAE has clear relevance for primary care practice, community-based screening, geriatric service planning, and family-centered caregiving models. Despite this, empirical data examining cognitive function, its relationship with QOL, and the influence of sociodemographic factors such as education, income, marital status, and living arrangements among older adults in the UAE remain limited.

Addressing this evidence gap is essential for informing context-specific strategies aimed at promoting healthy aging in the United Arab Emirates. Therefore, this study aims to assess cognitive function and its association with QOL among adults aged 60 years and older in the UAE, conceptualizing QOL as a multidimensional outcome reflecting functional ability, independence, and overall wellbeing. In addition, the study seeks to identify sociodemographic and health-related factors associated with QOL in this population, providing evidence to support targeted interventions and informed policy development for aging populations.

## Materials and methods

### Ethical considerations

The study received ethical approval from the Research Ethics Committee at the Ministry of Health and Prevention (MOHAP/DXB-REC/D21. J22. N22/No.136/2021) and from the Research Ethics Committee of the XXXX (REC-22–03-14). All procedures were conducted in accordance with the Declaration of Helsinki. Written informed consent was obtained from all participants. For individuals with cognitive impairment or inability to sign, verbal explanation and assent were obtained, and consent was confirmed with the assistance of a family member or social services representative. Participation was voluntary, confidentiality was maintained, and no incentives were provided. This project was funded by the XXXX Target Research Program (Grant project number: 2301050394).

### Study design, setting, and recruitment

This quantitative cross-sectional study was conducted among community-dwelling older adults residing in the United Arab Emirates. Participants were recruited using convenience sampling through social services departments in Dubai, outpatient clinics, and the Centre of Excellence for Dental Care for Older Adults at a university-affiliated hospital in Sharjah. Accordingly, Sharjah and Dubai were the main recruitment sites for this study. It is important to note that Emirati older adults in Sharjah have access to free medical insurance coverage for dental services, which contributed to a higher proportion of participants being recruited from the Centre of Excellence.

Coordination with these entities facilitated access to older adults receiving community-based health and social support services, including home care, rehabilitation, transportation assistance, and social engagement programs. Most participants were recruited through the Centre of Excellence.

Eligible individuals were approached by trained research assistants. Prior to data collection, the research assistants underwent structured training covering study objectives, standardized recruitment procedures, ethical considerations, informed consent processes, and consistent participant communication. The study objectives and procedures were then explained verbally to all eligible participants prior to enrolment.

### Eligibility criteria

Participants were eligible if they were aged 60 years or older, residing in the United Arab Emirates, able to communicate in Arabic or English, and functionally independent within a community setting. The study included all older adults residing in the UAE, both Emirati and non-Emirati, regardless of nationality, provided they were willing to participate and able to communicate in either Arabic or English. For expatriate participants, a minimum residency period of 1 year was required to ensure sufficient exposure to the UAE healthcare system and living environment, as shorter residency may influence QOL responses. Participants who were unable to communicate in either language or who declined participation were excluded. For those unable to read, interviewer-administered questionnaires were conducted by trained research assistants.

### Sample size calculation

The sample size was calculated using the single-population proportion formula (*n* = 4PQ/*d* ^2^), where *P* represents the estimated prevalence, *Q* = 1 − *P*, and *d* denotes the margin of error. An estimated prevalence of cognitive impairment of 50.2% was adopted based on a recent community-based study using the Montreal Cognitive Assessment among older adults (Tawfik et al., 2024). Assuming a 95% confidence level and a 5% margin of error, the minimum required sample size was approximately 384 participants. To compensate for potential non-response and incomplete data, the sample size was increased by 30%, yielding a target sample of approximately 500 participants. Ultimately, 606 eligible participants were recruited and included in the analysis. The larger final sample was retained to enhance statistical power, improve precision of estimates, and strengthen the representativeness and generalizability of the findings.

## Data collection instruments

### Sociodemographic, health, and lifestyle characteristics

The questionnaire collected information on age, sex, nationality, place of residence, marital status, living arrangement, educational attainment, employment status, monthly income, availability of home helpers, presence of chronic medical conditions, and health insurance coverage. Lifestyle-related variables included smoking status, engagement in regular physical activity, and self-rated dietary pattern. Body mass index (BMI) was calculated using direct measurements of weight in kilograms and height in meters.

### Quality of life assessment

QOL was assessed using the Older People’s Quality of Life Questionnaire–Brief version (OPQoL-Brief), a validated instrument developed to measure multidimensional QOL in older populations (Bowling et al., 2013). The instrument consists of 13 items rated on a five-point Likert scale, with higher scores indicating better perceived QOL. The total score ranges from 13 to 65 and encompasses four domains: wellbeing and life satisfaction, health and functioning, social relationships and participation, and financial circumstances. A single global quality-of-life item is included but not incorporated into the total score.

The OPQoL-Brief has demonstrated robust psychometric properties, including high internal consistency and construct validity across diverse older populations (Bowling et al., 2013). A validated Arabic version of the OPQoL-Brief was used for Arabic-speaking participants and has demonstrated excellent internal consistency (Cronbach’s α = 0.92) and good concurrent validity (Sousa and Rojjanasrirat, 2011). Minor linguistic and contextual adaptations were applied to ensure cultural appropriateness for use in the UAE without altering the conceptual meaning of the items. The QOL scale demonstrated acceptable internal consistency (Cronbach’s α = 0.756). The standardized Cronbach’s alpha was 0.914, indicating higher internal consistency when accounting for differences in item scaling.

### Cognitive function assessment

Cognitive function was assessed using the Montreal Cognitive Assessment (MoCA), a widely used 30-point screening tool designed to detect mild cognitive impairment and early cognitive decline (Nasreddine and Patel, 2023; Nasreddine et al., 2005). The MoCA evaluates multiple cognitive domains, including memory, attention, language, visuospatial and executive function, abstraction, and orientation. A score of 26 or above indicates normal cognitive performance, while lower scores suggest cognitive impairment (Nasreddine et al., 2005). The validated Arabic version of the MoCA was used in this study and has demonstrated good reliability and diagnostic accuracy among Arabic-speaking older adults, with reported Cronbach’s α of 0.83 and high sensitivity and specificity for detecting cognitive impairment (Ajrouch et al., 2024).

### Statistical analysis

Data were analyzed using IBM® SPSS® Statistics 30.0. Descriptive statistics were used to summarize participant characteristics, with categorical variables presented as frequencies and percentages, and continuous variables as means and standard deviations. Prior to inferential analyses, assumptions of normality were assessed and the distribution of variables was examined for potential floor and ceiling effects. Pearson correlation analysis was performed to examine associations between cognitive function scores and quality-of-life measures. Independent sample *t*-tests and one-way analysis of variance were used to compare mean QOL scores across sociodemographic and health-related groups.

To determine the appropriateness of using parametric testing, assumptions of normality were assessed. The normality of continuous variables was examined using the Kolmogorov–Smirnov and Shapiro–Wilk tests. Both tests indicated statistically significant deviations from normality (p < 0.001), which was expected given the large sample size (n = 606), as normality tests are highly sensitive and may detect trivial departures that are not practically meaningful in large datasets (Ghasemi and Zahediasl, 2012). Therefore, visual inspection of histograms and Q-Q plots was additionally performed, which demonstrated that the distribution of QOL scores was approximately normal, with no evidence of substantial skewness or outliers, supporting the appropriateness of parametric analyses (Field, 2024; Ghasemi and Zahediasl, 2012). This provides support for the reliability and validity of the statistical analyses and study findings. Researchers also compared the results of parametric and non-parametric tests, which yielded consistent and closely comparable findings, supporting the robustness of the statistical analyses.

When ANOVA indicated statistically significant differences between groups, *post hoc* pairwise comparisons were conducted using Tukey’s HSD test. Statistically significant differences were presented in the tables using superscript letters to facilitate interpretation of pairwise group differences (P < 0.05).

Multiple linear regression analysis was conducted to identify independent determinants of QOL, with the OPQoL-Brief total score as the dependent variable. Variables showing significant associations in univariate analyses were entered into the multivariable model. Model assumptions, including normality, linearity, and multicollinearity, were assessed prior to analysis. Variance Inflation Factor (VIF) values ranged from 1.05 to 3.56 indicating that multicollinearity is not a concern in the model and does not affect the stability or interpretation of the regression estimates (Hair Jr et al., 2010). A complete-case analysis approach was applied, and statistical significance was set at *p* < 0.05.

## Results

### Sociodemographic and general characteristics of the sample

Table 1 presents the sociodemographic, lifestyle, and health-related characteristics of the study population. A total of 606 participants were included, of whom 55.4% were male and 44.6% were female. The majority were Emirati (84.2%). Most participants resided in Sharjah (74.9%), while 25.1% lived in Dubai. Regarding educational attainment, 44.6% of participants had no formal education or had completed elementary education, 35.0% had completed high school or a diploma, and 20.5% held a university degree (BSc, MSc, or PhD). Most participants lived with their family (96.7%) and were married (76.6%). The majority were retired/currently not employed (88.1%).

**TABLE 1:** General Characteristics of participants (N = 606).

Variable	Category	Frequency	Percent
Sex	Male	336	55.4
	Female	270	44.6
Age	60–63 years	244	40.3
	64–66 years	166	27.4
	67–87 years	196	32.3
Nationality	Non-emirati	96	15.8
	Emirati	510	84.2
Residency	Dubai	152	25.1
	Sharjah area (Kalba, Khor Fakkan, Dibba Al-Hisn	454	74.9
Education	Unable to read and elementary	270	44.6
	High school and a diploma	212	35.0
	BSc, MSc, PhD	124	20.5
Live with who	Alone	20	3.3
	Family	586	96.7
Marital status	Single, divorced, widowed	142	23.4
	Married	464	76.6
Employment	Not employed	534	88.1
	Employed	72	11.9
Average income	5K–15K	168	27.7
	15K–25K	320	52.8
	More than or equal to 25K	118	19.5
Helper	No	94	15.5
	Yes	512	84.5
Health insurance	No	62	10.2
	Yes	544	89.8
Chronic medical condition	No	82	13.5
	Yes	524	86.5
*BMI (kg/M^2^)	Normal weight	116	19.1
	Overweight	208	34.3
	Obese	282	46.5
Regular physical activity (e.g., walking, exercise, sports) at least 3–5 times per week?	No	242	39.9
	Yes	364	60.1
Smoking status	No	559	92.2
	Yes	47	7.8
Rating the overall dietary pattern	Poor	26	4.3
	Fair	77	12.7
	Good	503	83.0

* **BMI classifications** based on WHO standards. underweight (<18.5), normal weight (18.5–24.9), overweight (25.0–29.9), and obese (≥30.0). (World Health Organization. (2022). *Obesity and overweight*. https://www.who.int/news-room/fact-sheets/detail/obesity-and-overweight).

Reported monthly household income ranged from 5,000 to 15,000 AED in 27.7% of participants, 15,000 to 25,000 AED in 52.8%, and 25,000 AED or more in 19.5%. Most participants reported having a helper at home (84.5%) and having health insurance coverage (89.8%). Chronic medical conditions were reported by 86.5% of participants. Based on BMI classification, 19.1% had normal weight, 34.3% were overweight, and 46.5% were classified as obese. Regular physical activity was reported by 60.1% of participants. Most participants were non-smokers (92.2%). Overall dietary pattern was rated as good by 83.0% of participants, fair by 12.7%, and poor by 4.3%.

### Cognitive function measured by the Montreal Cognitive assessment

The Montreal Cognitive Assessment was administered to all 606 participants to evaluate cognitive function across seven domains (Table 2). The mean total MoCA score was 21.3 ± 6.0, with scores ranging from 5 to 30. Based on established cutoffs, 30.7% of participants scored 26 or higher, indicating normal cognitive function, while 69.3% scored below 26, indicating cognitive impairment. Among those with cognitive impairment, 40.9% had mild impairment with scores between 18 and 25, 24.8% had moderate impairment with scores between 10 and 17, and 3.6% had severe impairment with scores below 10. At the domain level, the highest mean scores were observed for orientation (mean 5.7 ± 0.7) and naming (mean 2.7 ± 0.6). Lower mean scores were observed for abstraction (mean 1.5 ± 0.8). Mean scores for memory, language, attention, and visuospatial or executive function domains were intermediate.

**TABLE 2:** MOCA total and domain scores (n = 606).

MOCA domains	Description	Marks	Mean	S.D.	Minimum achieved score	Maximum achieved score
Domain 1: Visuospatial executive	• Trail making • Cube copy • Clock drawing	5	3.0	1.7	0.0	5.0
Domain 2: Naming	• Animal naming	3	2.7	0.6	0.0	3.0
Domain 3: Attention	1. Digit span (forward and backward) 2. Vigilance 3. Serial 7 subtraction	6	4.1	2.0	0.0	6.0
Domain 4: Language	• Sentence repetition • Fluency	3	1.7	1.0	0.0	3.0
Domain 5: Abstraction	• Similarity pairs	2	1.5	0.8	0.0	2.0
Domain 6: Memory	• 5-Word recall test (2 learning trials • Delayed recall)	5	2.6	1.8	0.0	5.0
Domain 7: Orientation	• Date and location	6	5.7	0.7	1.0	6.0

Total MOCA raw score		30	21.3	6.0	5.0	30.0
High MOCA score (no cognitive problem) (≥26/30)	186 (30.7)	​	​	​	​	​
Low MOCA score (suggesting cognitive problem) (<26/30)	420 (69.3)	Mild cognitive impairment, 18–25 points			248 (40.9)	
		Moderate cognitive impairment, 10–17 points			150 (24.8)	
		Severe cognitive impairment, under 10 points			22 (3.6)	

### Quality of life measured by the OPQoL-Brief questionnaire

Tables 3, 4 present item-level responses, domain scores, and total scores for the OPQoL-Brief questionnaire. Mean item scores ranged from 3.58 ± 1.14 for social or leisure activities to 4.56 ± 0.58 for feelings of safety and pleasure derived from home. For most items, the majority of participants selected “agree” or “strongly agree” responses, indicating a ceiling effect in the QoL variables. This resulted in reduced score variability across items. The mean score for the single-item global QOL assessment was 3.25 ± 0.75, with 86.8% of participants rating their overall QOL as good or very good. Domain-level mean scores were 24.98 ± 3.40 for wellbeing, 8.67 ± 1.42 for health, 16.67 ± 2.27 for social relationships and participation, and 4.44 ± 0.67 for financial circumstances. The mean total OPQoL-Brief score was 54.76 ± 6.68, with scores ranging from 27 to 65. Based on the median cutoff, 49.5% of participants were classified as having higher QOL.

**TABLE 3:** Quality of life scores for participants (N = 606).

Item	Question/Item	M±S.D.#	Min	Max	Distribution of responders n (%)		
​	​	​	​	​	D/SD*	Nr*	A/SA*
QOL.1	Enjoy my life overall	4.19 ± 0.80	2.0	5.0	42 (6.9)	22 (3.6)	542 (89.4)
QOL.2	Look forward to things	3.88 ± 0.92	2.0	5.0	78 (12.9)	62 (10.2)	466 (76.9)
QOL.3	Healthy enough to get out and about	4.30 ± 0.82	1.0	5.0	38 (6.3)	14 (2.3)	554 (91.4)
QOL.4	Help from family, friends, or neighbors	4.11 ± 0.79	1.0	5.0	42 (6.9)	22 (3.6)	542 (89.4)
QOL.5	Have social or leisure activities/hobbies	3.58 ± 1.14	1.0	5.0	162 (26.7)	44 (7.3)	400 (66.0)
QOL.6	Staying involved with things	3.92 ± 0.84	2.0	5.0	56 (9.2)	74 (12.2)	476 (78.5)
QOL.7	Healthy enough to have my independence	4.37 ± 0.71	2.0	5.0	22 (3.6)	16 (2.6)	568 (93.7)
QOL.8	Please myself with what I do	4.30 ± 0.68	2.0	5.0	18 (3.0)	22 (3.6)	566 (93.4)
QOL.9	Feel safe where I live	4.56 ± 0.57	2.0	5.0	6 (1.0)	6 (1.0)	594 (98.0)
QOL.10	Get pleasure from my home	4.56 ± 0.58	2.0	5.0	8 (1.3)	4 (0.7)	594 (98.0)
QOL.11	Take life as it comes and make the best of things	4.22 ± 0.69	2.0	5.0	10 (1.7)	62 (10.2)	534 (88.1)
QOL.12	Feel lucky compared to most people	4.20 ± 0.83	1.0	5.0	42 (6.9)	26 (4.3)	538 (88.8)
QOL.13	Have enough money to pay for household bills	4.44 ± 0.67	1.0	5.0	16 (2.6)	6 (1.0)	584 (96.4)

Single-item global assessment		M±S.D.#	Min	Max	B/VB*	Ar*	G/VG*
QOL.SIGA	Rate overall QOL? *(not included in the total score and the domains’ score)*	3.25 ± 0.75	0.0	4.0	10 (1.7)	70 (11.6)	526 (86.8)

M ± S.D., Mean ± Standard deviation. D/SD, disagree/strongly disagree; Nr = neither; A/SA, agree/strongly agree. B/VB, bad/very bad; G/VG, good/very good; Ar. = Alright. SIGA, general assessment.

**TABLE 4:** Quality of life domain scores (N = 606).

Domains	Items	M ± S.D.#	Min	Max
Wellbeing domain	Q1, Q2, Q6, Q10, Q11, Q12	4.16 ± 0.57	2.17	5.0
Health domain	Q3, Q7	4.33 ± 0.71	1.5	5.0
Social relation and participation	Q4, Q5, Q8, Q9	4.13 ± 0.55	1.5	5.0
Financial circumstances domain	Q13	4.44 ± 0.67	1.0	5.0
**QOL scores**		​	​	​
QOL total score		54.76 ± 6.68	27.0	65.0
QOL mean score		4.21 ± 0.51	2.08	5.0
**QOL classification (median = 54.0)**	n (%)			
Higher QOL (≥median)	300 (49.5)			
Lower QOL (<median)	306 (50.5)			

### Associations between cognitive function, sociodemographic factors, and quality of life

Table 5 presents Pearson correlation coefficients between MoCA scores and QOL measures. Total MoCA scores were positively and significantly correlated with the total OPQoL-Brief score, all QOL domains, and the single-item global QOL assessment. All MoCA cognitive domains were significantly correlated with the total QOL score and with the wellbeing, health, and social relationship domains. Correlations with the financial circumstances domain were weaker but remained statistically significant for most cognitive domains. The single-item global QOL assessment was significantly correlated with total MoCA scores and with most cognitive domains, except memory. Supplementary Table 1 presents univariate analyses of total QOL scores across sociodemographic and lifestyle variables. Significant differences in mean QOL scores were observed across several sociodemographic characteristics. Post hoc analyses (Tukey HSD/Bonferroni) showed that participants aged 67–87 years had significantly lower QOL scores compared to those aged 60–63 and 64–66 years (p < 0.05), with no significant difference between the two younger groups. For education, participants with lower educational attainment (unable to read/elementary) had significantly lower QOL scores than those with high school/diploma and higher education (p < 0.001), while no significant difference was observed between high school/diploma and higher education levels. For income, QOL scores increased significantly across all categories, with participants earning ≥25K reporting the highest scores, followed by 15K–25K and 5K–15K (all p < 0.001). No significant differences were observed for nationality, residency, health insurance status, body mass index category, smoking status, or dietary pattern.

**TABLE 5:** Correlation between QOL total and–MOCA total and subdomains (n = 606) [Pearson’s *r (P-value)]*.

Variable	Total QOL score	QOL domains				Single-item global assessment
		Wellbeing domain	Health domain	Social relation and participation	Financial circumstances domain	
Total MOCA score	.365** (<0.001)	.338** (<0.001)	.325** (<0.001)	.310** (<0.001)	.184** (<0.001)	.241** (<0.001)
MOCA domain 1: Visuospatial executive	.276** (<0.001)	.277** (<0.001)	.266** (<0.001)	.200** (<0.001)	.102* (0.012)	.226** (<0.001)
MOCA domain 2: Naming	.225** (<0.001)	.204** (<0.001)	.175** (<0.001)	.216** (<0.001)	.107** (0.008)	.124** (0.002)
MOCA domain 3: Attention	.295** (<0.001)	.285** (<0.001)	.273** (<0.001)	.235** (<0.001)	.116** (0.004)	.214** (<0.001)
MOCA domain 4: Language	.292** (<0.001)	.292** (<0.001)	.230** (<0.001)	.239** (<0.001)	.134** (0.001)	.199** (<0.001)
MOCA domain 5: Abstraction	.276** (<0.001)	.268** (<0.001)	.248** (<0.001)	.227** (<0.001)	.096* (0.018)	.212** (<0.001)
MOCA domain 6: Memory	.216** (<0.001)	.153** (<0.001)	.175** (<0.001)	.236** (<0.001)	.208** (<0.001)	.060 (0.156)
MOCA domain 7: Orientation	.170** (<0.001)	.153** (<0.001)	.177** (<0.001)	.137** (0.001)	.080* (0.050)	.152** (<0.001)

p < 0.05 (*), p < 0.01 (**).

### Determinants of quality of life

Table 6 presents the results of the multiple linear regression analysis examining factors associated with total QOL scores among 606 older adults. The multiple linear regression model was statistically significant, F(22, 583) = 9.494, P < 0.001. The model explained 26.4% of the variance in QoL (*R* ^2^ = 0.264; adjusted *R* ^2^ = 0.236), with R = 0.514 and a standard error of the estimate of 5.83, indicating a moderate level of explanatory power.

**TABLE 6:** The determinants of the QOL among the older adults using multiple linear regression (n = 606).

Variable	Multiple linear regression		VIF
	β (95%CI)	P value	
Sex [ref: Male]			
Female	−2.093 (−3.193, −0.992)	<0.001	1.381
Age [ref: 60 to 63 years]			
64–66 years	0.280 (−0.790, 1.350)	0.610	1.317
67–87 years	−1.620 (−2.660, −0.580)	0.002	1.400
Nationality [ref: non-emirati]			
Emirati	0.105 (−1.638, 1.847)	0.906	1.891
Residency [ref: Dubai]			
Sharjah	0.358 (−0.784, 1.500)	0.539	1.122
Education [ref: Unable to read and elementary]			
High school and a diploma	3.170 (2.050, 4.290)	<0.001	1.423
BSc, MSc, PhD	4.770 (3.460, 6.080)	<0.001	1.972
Live with who [ref: alone]			
Family	2.920 (0.048, 5.792)	0.046	1.205
Marital status [ref: Single, divorced, widowed]			
Married	1.610 (0.287, 2.934)	0.017	1.482
Employment [ref: Not employed]			
Employed	1.435 (−0.382, 3.252)	0.121	1.618
Income [ref: 5K to 15K]			
15K–25K	2.970 (1.800, 4.140)	<0.001	1.845
More than or equal to 25K	6.650 (5.240, 8.060)	<0.001	1.920
Helper [ref: No]			
Yes	−0.722 (−2.314, 0.871)	0.374	1.550
Health insurance [ref: No]			
Yes	1.331 (−0.409, 3.072)	0.134	1.279
Chronic medical medication [ref: No]			
Yes	−1.312 (−2.745, 0.120)	0.073	1.127
BMI [ref: Normal weight]			
Overweight	0.560 (−0.770, 1.890)	0.410	1.872
Obese	−0.420 (−1.780, 0.940)	0.550	1.895
Regular physical activity [ref: No]			
Yes	2.173 (1.190, 3.155)	<0.001	1.062
Smoking status [ref: No]			
Yes	−1.001 (−2.788, 0.785)	0.271	1.046
Rating the overall dietary pattern [ref: Fair]			
Poor	0.200 (−2.380, 2.780)	0.880	3.555
Good	−0.770 (−2.320, 0.780)	0.330	3.533
MOCA cutoff [ref: Normal MoCA]			
Below a normal MOCA score (cognitive problem)	0.138 (0.043, 0.232)	0.001	1.471

Significant values are bold. The QOL, total score was entered into the regression model as a dependent variable. The overall model was statistically significant, F(22, 583) = 9.494, P < 0.001, R2 = 0.264; adjusted R2 = 0.236. VIF, is the Variance Inflation Factor.

Several sociodemographic characteristics were independently associated with higher QOL after adjustment for all covariates. Female participants reported significantly lower QOL scores compared with males (β = −2.093; 95% CI: −3.193, −0.992; *P* < 0.001). Advancing age was also associated with reduced QOL, with participants aged 67–87 years scoring lower than younger age groups. Higher educational attainment showed a strong positive association with QOL. Compared with the reference group, participants with high school or diploma education (β = 3.170; 95% CI: 2.050, 4.290; *P* < 0.001) and those holding a university degree (BSc, MSc, or PhD) (β = 4.770; 95% CI: 3.460, 6.080; *P* < 0.001) reported significantly higher QOL scores. Living with family and being married were also independently associated with higher QOL (β = 2.920; 95% CI: 0.048, 5.792; *P* = 0.046 and β = 1.610; 95% CI: 0.287, 2.934; *P* = 0.017, respectively). Monthly household income demonstrated a graded positive association with QOL. Participants earning 15,000–25,000 AED (β = 2.970; 95% CI: 1.800, 4.140; *P* < 0.001) and ≥25,000 AED (β = 6.650; 95% CI: 5.240, 8.060; *P* < 0.001) reported significantly higher QOL compared with those in the lowest income category. Regular physical activity was similarly associated with higher QOL (β = 2.173; 95% CI: 1.190, 3.155; *P* < 0.001).

In contrast, nationality, place of residence, employment status, presence of a home helper, health insurance coverage, chronic medication use, body mass index category, smoking status, and overall dietary pattern were not significantly associated with QOL (all *P* > 0.05). Importantly, better cognitive function (i.e., no cognitive impairment based on the MoCA cutoff) was independently associated with higher QoL scores compared with cognitive impairment (β = 0.138; 95% CI: 0.043–0.232; P = 0.001).

## Discussion

The study findings highlight the multidimensional nature of quality of life (QoL) in older adults in the UAE, with cognitive function emerging as a key determinant alongside important sociodemographic and lifestyle factors. In particular, poorer cognitive performance was significantly associated with lower QoL, underscoring its central role in shaping overall wellbeing in later life. The findings provide robust evidence to inform healthcare policy in the UAE, emphasizing the need for an integrated, person-centered approach to healthy aging that incorporates early cognitive screening while also addressing broader social and lifestyle determinants of health outcomes.

A key finding of this study is the high prevalence of cognitive decline, with 69.3% of participants scoring below the Montreal Cognitive Assessment cutoff of 26. Specifically, 40.9% were classified with mild cognitive impairment, and 3.6% had scores that may suggest more severe impairment. It is important to note that the MoCA is a screening instrument and scores suggestive of dementia do not represent a clinical diagnosis. These rates are comparable to recent community-based evidence in the Arab region, including high prevalence estimates in Egypt and Lebanon (Tawfik et al., 2024; Karam et al., 2024). Regional cost-of-illness analyses further highlight the burden of cognitive disorders among older adults in North Africa and the Middle East (Qassem et al., 2023).

Participants scored lowest in abstraction, consistent with evidence identifying higher-order cognitive processes as particularly vulnerable in aging populations. Abstraction is related to executive function and reasoning, which recent research has linked with life satisfaction and independence among older adults (Oh and Yang, 2021; Laera et al., 2021). Emerging evidence suggests that structured cognitive training that includes analogy tasks, reasoning exercises, and culturally relevant group activities may enhance executive processes and support cognitive health (Rodriguez-Rodríguez et al., 2024; Castellote-Caballero et al., 2024).

In the UAE context, participants generally reported moderate-to-high QOL, with the highest satisfaction in financial and health domains. This likely reflects the influence of national and emirate-level support systems that promote independence and access to services. Community support has been shown to promote life satisfaction and wellbeing in older populations (Shen et al., 2022). Despite these supports, lower scores in social participation highlight gaps in meaningful engagement in leisure and hobby activities, which newer evidence shows are linked with sustained QOL and reduced cognitive decline (Robertson et al., 2023).

Our results demonstrate significant positive associations between global cognitive function and multiple QOL domains, including wellbeing, health, social participation, and financial security. These findings align with recent large-scale studies indicating that preserved cognitive function supports functional independence and overall wellbeing (Ye et al., 2025; Mendorf et al., 2025), and those cognitive deficits negatively affect health-related QOL (Hui et al., 2025). Even after adjustment for sociodemographic and lifestyle variables, cognitive impairment remained independently associated with lower QOL, highlighting its critical role in healthy aging.

Routine cognitive assessment in community settings may facilitate early detection of mild cognitive impairment, enabling earlier interventions that support autonomy and social functioning (Fowler et al., 2025). Telehealth and digital approaches to cognitive assessment have gained prominence, with recent evidence supporting their reliability and scalability in older populations (Grueso and Viejo-Sobera, 2021). Artificial intelligence-assisted tools may further enhance sensitivity to subtle changes in cognitive performance, enabling personalized recommendations and preventive strategies (Li et al., 2022).

Sociodemographic determinants of QOL in this study mirror patterns reported internationally. Female participants and older age groups exhibited lower QOL, whereas higher education, living with family, marital status, and higher income were associated with better outcomes (Llorens-Ortega et al., 2024). Regular physical activity was also a positive determinant, aligning with recent meta-analytic evidence showing that greater physical activity intensity is associated with improved cognitive and functional outcomes in aging populations (Chang et al., 2025). Physical activity may also mediate the association between aging, cognitive function, and QOL, buffering decline and promoting sustained wellbeing (Lin et al., 2025; Hui et al., 2025).

Collectively, these results highlight that QOL in older adulthood is multifactorial, resulting from the interplay among cognitive health, social engagement, lifestyle behaviors, and sociodemographic resources. Understanding these interactions can inform culturally tailored interventions that integrate cognitive, physical, and social domains to enhance wellbeing in later life and guide public health strategies in the UAE.

### Strength and limitations

This study has several strengths. It includes a relatively large community-based sample of older adults in the UAE, enhancing the robustness and real-world relevance of the findings. The use of a validated quality of life instrument (OPQoL-Brief) and a standardized cognitive assessment tool (MoCA) strengthens the reliability and validity of the measurements. In addition, the study protocol ensured standardized data collection procedures through trained research assistants, which helped reduce measurement bias and improve data quality. Furthermore, the simultaneous examination of cognitive function alongside a broad range of sociodemographic and lifestyle factors provides a more comprehensive and contextually relevant understanding of independent determinants of QOL in older adults.

This study primarily included Emirati older adults, with limited representation of expatriate older adult populations who may differ in social, cultural, and economic contexts that influence cognitive function and QOL, potentially limiting the generalizability of the findings to the broader aging population in the UAE. The cross-sectional design precludes assessment of temporal relationships or causal pathways between cognitive function and QOL. In addition, lifestyle and health-related variables were self-reported and may be subject to reporting or recall bias. Another limitation of this study is that participants were recruited only from two emirates (Sharjah and Dubai), which may limit the generalizability of the findings to the broader older adult population across all seven emirates of the United Arab Emirates.

While the Montreal Cognitive Assessment is widely used as a screening tool, region-specific normative cutoff values for older adults in the UAE have not yet been established. Future research should employ longitudinal designs, incorporate pathway or mediation analyses, and validate culturally adapted cognitive assessment tools to improve detection accuracy and to better elucidate the direct and indirect effects of cognitive decline on QOL. A potential ceiling effect was present in the QoL measure, as many participants reported high scores across items, which may have reduced variability in the outcome and consequently limited the sensitivity to detect associations or group differences.

## Conclusion

The study objectives were achieved, demonstrating a significant association between cognitive function and quality of life (QoL) among community-dwelling older adults in the UAE, with better cognitive function independently associated with higher QoL. In addition, QoL was influenced by multiple sociodemographic and lifestyle factors, including age, sex, education, income, marital status, living arrangements, physical activity, and medical conditions. Overall, these findings indicate that QoL in later life is shaped by an interplay of cognitive, social, and lifestyle determinants.

The findings underscore the importance of early cognitive screening, promotion of physical activity, and support for social and financial wellbeing as key strategies to enhance QoL in later adulthood. These results provide evidence to guide targeted interventions and inform age-friendly policies that adopt a holistic, person-centred approach. Future research should focus on elucidating the mechanisms through which cognitive function influences QoL and identifying modifiable factors that may mitigate age-related decline, thereby supporting the development of effective preventive strategies for healthy aging.

## Data Availability

The raw data supporting the conclusions of this article will be made available by the authors, without undue reservation.
