# Supplementary material for: Cognitive decline, and quality of life: evidence from community-dwelling older adults in the United Arab Emirates
Source: Front Aging. 2026 May 29;7:1822042. doi: 10.3389/fragi.2026.1822042 (PMC13260358; doi:10.3389/fragi.2026.1822042)
Supplement: Supplementary file 1 [file Table1.docx]

**Supplementary Table 1: Univariate analysis (ANOVA and T-test) for total QOL total score with the demographic data (n=606)**

| Variable | Category | (Mean ± S.D.) | P-value |
| --- | --- | --- | --- |
| Sex# | Male | 56.50 ± 5.79 | <0.001* |
|  | Female | 52.59 ± 7.07 |  |
| Age* | 60 to 63 years | 55.20 ± 6.36 ^a^ | 0.010* |
|  | 64 to 66 years | 55.48 ± 6.11 ^a^ |  |
|  | 67 to 87 years | 53.58 ± 7.35 ^b^ |  |
| Nationality# | Non-Emirati | 55.94 ± 6.70 | 0.059 |
|  | Emirati | 54.53 ± 6.65 |  |
| Residency # | Dubai | 54.75 ± 6.20 | 0.990 |
|  | Sharjah | 54.76 ± 6.83 |  |
| Education* | Unable to read and elementary | 52.67 ± 6.76 ^a^ | <0.001* |
|  | High school and a diploma | 55.84 ± 6.03 ^b^ |  |
|  | BSc, MSc, PhD | 57.44 ± 6.18 ^b^ |  |
| Live with who# | Alone | 49.10 ± 8.89 | <0.001* |
|  | Family | 54.95 ± 6.51 |  |
| Marital status# | Single, divorced, widowed | 51.72 ± 7.59 | <0.001* |
|  | Married | 55.69 ± 6.08 |  |
| Employment# | Not employed | 54.25 ± 6.67 | <0.001* |
|  | Employed | 58.47 ± 5.46 |  |
| Income* | 5K to 15K | 51.89 ± 7.12 ^a^ | <0.001* |
|  | 15K to 25K | 54.86 ± 6.16 ^b^ |  |
|  | More than or equal to 25K | 58.54 ± 5.32 ^b^ |  |
| Helper# | No | 56.02 ± 6.64 | 0.045* |
|  | Yes | 54.52 ± 6.66 |  |
| Health insurance# | No | 53.58 ± 6.38 | 0.144 |
|  | Yes | 54.89 ± 6.70 |  |
| Chronic Medical condition# | No | 56.32 ± 5.05 | 0.023* |
|  | Yes | 54.51 ± 6.87 |  |
| BMI* | Normal weight | 54.76 ± 6.54 | 0.278 |
|  | Overweight | 55.32 ± 6.12 |  |
|  | Obese | 54.34 ± 7.10 |  |
| Regular physical activity# | No | 53.10 ± 7.06 | <0.001* |
|  | Yes | 55.86 ± 6.18 |  |
| Smoking status# | No | 54.62 ± 6.72 | 0.086 |
|  | Yes | 56.36 ± 5.99 |  |
| Rating the overall dietary pattern* | Fair | 54.62 ± 6.49 | 0.755 |
|  | Poor | 54.82 ± 6.74 |  |
|  | Good | 53.85 ± 6.11 |  |
| MOCA total | Normal MOCA score (Normal cognition) | 57.18 ± 6.31 | <0.001* |
|  | Low MOCA score (Cognitive problem) | 53.68 ± 6.56 |  |

^*ANOVA. #Independent Sample t-test. Cognitive problem < 26 out of 30. Different superscript letters indicate statistically significant differences (Tukey post-hoc test, P < 0.05)^
